# Impact of new regimens and drugs on rifampin-resistant tuberculosis management in Mexico

**DOI:** 10.36416/1806-3756/e20250131

**Published:** 2025-11-19

**Authors:** Marcela Muñoz-Torrico, Rafael Laniado-Laborín, Jorge Rojas-Serrano, Eduardo Becerril-Vargas, Wendy Cinecio-Chávez, Fátima Leticia Luna-López, Luis Armando Narvaez-Díaz, Roberto Rentería-Gamez, Mariela Segura del Pilar, Nallely Saavedra, Julio César Magaña, Lia D’Ambrosio, Rosella Centis, José Antonio Caminero, Giovanni Battista Migliori

**Affiliations:** 1. Clínica de Tuberculosis, Instituto Nacional de Enfermedades Respiratorias Ismael Cosío Villegas, Ciudad de México, México.; 2. Facultad de Medicina y Psicología, Universidad Autónoma de Baja California, Tijuana, México.; 3. Departamento de Reumatología, Instituto Nacional de Enfermedades Respiratorias Ismael Cosío Villegas, Ciudad de México, México.; 4. Laboratorio de Microbiología Clínica, Instituto Nacional de Enfermedades Respiratorias Ismael Cosío Villegas, Ciudad de México, México.; 5. Programa Nacional de Tuberculosis y Micobacteriosis, Centro Nacional de Prevención y Control de Enfermedades - CENAPRECE - Ciudad de México, México.; 6. Public Health Consulting Group, Lugano, Switzerland.; 7. Servizio di Epidemiologia Clinica delle Malattie Respiratorie, Istituti Clinici Scientifici Maugeri - IRCCS - Tradate, Italia.; 8. Servicio Neumología, Hospital Universitario de Gran Canaria Dr. Negrín, Las Palmas, España.; 9. Departamento de Actividades Científicas, Alosa TB Academy, Las Palmas, España.

**Keywords:** Mexico, Tuberculosis, multidrug-resistant, Bedaquiline, Linezolid, Treatment outcome

## Abstract

**Objective:**

To compare the former tuberculosis treatment regimen including one fluoroquinolone (ofloxacin, levofloxacin, or moxifloxacin) and a second-line injectable drug (amikacin, kanamycin, or capreomycin) plus three to five oral drugs (regimen 1) with the current regimen including the three WHO group A drugs (regimen 2) in terms of efficacy and safety at two tuberculosis referral centers in Mexico.

**Methods:**

This was a retrospective study based on a review of the clinical records of all consecutive rifampin-resistant or multidrug-resistant tuberculosis (RR/MDR-TB) patients treated from January of 2010 to October of 2023. Patients included were microbiologically confirmed cases of RR/MDR-TB with pulmonary involvement and who received at least 30 days of regimen 1 or regimen 2. Outcomes and adverse events were classified in accordance with WHO definitions.

**Results:**

One hundred and twenty-six RR/MDR-TB patients met the inclusion criteria. Of those, 87 were treated with regimen 1 and 39 received regimen 2. Success rates were not significantly different between the two groups of patients, although those treated with the oral regimen including bedaquiline from regimen 2 had higher success rates. Regimen 2 patients experienced a shorter time to culture conversion, and the regimen length was shortened accordingly, the median duration being 16.1 months [IQR, 15-17.3 months]. In patients receiving the all-oral regimen 2, adverse events were significantly associated with a history of type 2 diabetes mellitus (OR = 15.4; 95% CI, 2.73-87.29; p = 0.002) and were mainly related to linezolid use.

**Conclusions:**

Oral regimens appear to be effective, although toxicity to linezolid requires strict patient monitoring.

## INTRODUCTION

Drug-resistant tuberculosis remains a public health concern, particularly in Mexico, where the number of cases of rifampin-resistant or multidrug-resistant tuberculosis (RR/MDR-TB) in 2023 was estimated at 1,300 (range, 0-2,700), although only 444 were reported.^1^ During the last decade, significant progress has been achieved on tuberculosis diagnosis and treatment.^2^ ^-^ ^4^ Since 2019 (after the release of the STREAM (*Standardized Treatment Regimen of Anti-Tuberculosis Drugs for Patients with MDR-TB*) stage 1 study results, the WHO has recommended the use of a shorter (nine-month) regimen for the treatment of selected cases of RR/MDR-TB.^2^ ^-^ ^4^ Nevertheless, the availability of new oral drugs (e.g., bedaquiline) and repurposed drugs (e.g., fluoroquinolones, linezolid, and clofazimine) allowed the WHO to develop a new classification of antituberculosis drugs (groups A, B, and C) on the basis of their effectiveness and safety.^3^ ^,^ ^5^ The WHO approval of the all-oral six-month combinations of bedaquiline, pretomanid, and linezolid, with or without moxifloxacin, i.e., the BPaL/BPaLM regimens,^6^ opened new perspectives in the treatment of RR/MDR-TB. However, not all national tuberculosis programs, including the Mexican National Tuberculosis Program, have been able to implement the BPaL/BPaLM regimens (Table 1). 

**Table 1:** Comparison of sociodemographic and clinical characteristics of tuberculosis patients enrolled to receive treatment regimen 1 or 2.^a^

Variable	Regimen 1 (n = 87)	Regimen 2 (n = 39)	p
Male	59 (67.8%)	22 (56.4%)	0.217
Age, years	42 [33-55]	37 [28-50]	0.3924
Type 2 diabetes mellitus Disease duration Glucose at diagnosis, mg/dL^b^ Glycated hemoglobin at diagnosis, %^c^	43 (49.4%) 10 [7-14] 178 [134-252] 9.3 [7.9-10.9]	14 (35.9%) 9.5 [6.5-19] 186 [142-242] 9.4 [7.2-9.9]	0.158 0.8284 0.9883 0.3819
HIV infection CD4 count at diagnosis, cells/mm^3^	3/86 (3.5%) 88 [21-316]	7 (18%) 62.5 [30-111]	0.006 0.7963
Malnutrition (BMI < 18.5 kg/m^2^)	26/85 (30.6%)	9 (23.1%)	0.388
BMI, kg/m^2^	20.9 [18.2-24.7]	22 [19.6-24]	0.6551
Hypertension	13/86 (15.1%)	6 (15.4%)	0.969
Chronic kidney disease	11/85 (13%)	2 (5.1%)	0.187
Smoking history	7/28 (25%)	9 (23.1%)	0.856
History of drug abuse	8 (9.2%)	5 (12.8%)	0.536
Previous tuberculosis treatment	76 (87.3%)	21 (53.8%)	0.000
Weight, kg	55 [48-66]	56 [49.5-62.5]	0.6933
Hemoglobin, g/dL^d^	12.4 [10.8-14.1]	11.4 [10.5-13]	0.0945
Lymphocyte count, cells/µL	1.4 [1.3-2]	1.7 [0.9-2.1]	0.9470
Albumin, g/dL^d^	3.3 [2.9-3.7]	3.2 [2.9-3.6]	0.6541
Smear positive at diagnosis	73/86 (84.9%)	23/36 (63.9%)	0.010
Culture positive at diagnosis	85/86 (98.8%)	32 (82%)	0.001
RR^e^ MDR Pre-XDR^f^	7 75 5	12 26 1	
Chest X-ray Non cavities Unilateral cavities Bilateral cavities	86/87^g^ 15 (17.4%) 38 (44.2%) 33 (38.4%)	39 16 (41%) 13 (33.3%) 10 (25.6%)	0.018
Cavitary disease	71/86 (82.6%)	23 (59%)	0.005

RR: rifampin resistant; MDR: multidrug resistant (i.e., resistant to rifampin and isoniazid); and pre-XDR: pre-extensively drug resistant (i.e., MDR plus additional resistance to a fluoroquinolone). ^a^Data presented as n, n (%), or median [IQR]. ^b^Data available for 48 patients. ^c^Data available for 49 patients. ^d^Data available for 97 patients. ^e^5 patients with additional resistance to pyrazinamide (1 receiving regimen 1 and 4 receiving regimen 2). ^f^For regimen 1, 4 patients were resistant to ofloxacin and 1 patient was resistant to ofloxacin and moxifloxacin. For regimen 2, 1 patient was resistant to levofloxacin. ^g^Two patients had pleural involvement: 1 receiving regimen 1 and 1 receiving regimen 2.

Before the WHO reclassification of drugs, the standard regimen for RR/MDR-TB cases included one fluoroquinolone and a second-line injectable drug. After the reclassification, the longer regimen including the three group A drugs (levofloxacin or moxifloxacin, bedaquiline, and linezolid) and one group B drug (clofazimine and/or cycloserine)^7^ became the standard treatment for RR/MDR-TB cases in Mexico (Table 1). The WHO shorter regimens (of 9-11 months) initially including the use of an injectable drug (and later bedaquiline) were used in very few selected cases for different reasons, including the drug resistance profile of RR/MDR-TB patients in Mexico^8^ and the concern raised by the high number of drugs in these regimens, as well as their toxicity and potential impact on treatment adherence. 

Given the rapid evolution of regimens and the different approaches followed by countries to adopt the WHO recommendations, in-depth analyses of the effectiveness and safety of the longer all-oral regimens at the programmatic level are scanty.^9^

The objective of the present study was to compare the former regimen including one fluoroquinolone and a second-line injectable drug (regimen 1) with the current regimen including the three group A drugs (regimen 2) in terms of efficacy and safety at two tuberculosis referral centers in Mexico. 

## METHODS

### Study design

This was a retrospective study based on a review of the clinical records of all consecutive RR/MDR-TB patients treated between January of 2010 and October of 2023 at either of two tuberculosis referral centers in Mexico, namely, the *Instituto Nacional de Enfermedades Respiratorias* (INER), located in Mexico City, and the *Hospital General de Tijuana*, located in the city of Tijuana. The study was approved by the local research ethics committees. The requirement for informed consent was waived because of the retrospective nature of the study. 

### Diagnosis

The nationwide programmatic treatment of drug-resistant tuberculosis in Mexico started in 2010, when all presumptive drug-resistant patients were referred to tuberculosis referral centers, such as the INER and the *Hospital General de Tijuana*. Before the introduction of GeneXpert MTB/RIF in 2016, all cases were diagnosed by culture and phenotypic drug susceptibility tests, which were carried out in national referral laboratories. All laboratory procedures were (and still are) conducted in accordance with international guidelines, and drug susceptibility testing is performed using the critical concentrations suggested by the WHO.^10^ ^,^ ^11^

### Treatment

Second-line drugs in Mexico are provided by the Mexican National Tuberculosis Program, all cases being treated in accordance with WHO guidelines and drug susceptibility test results. Before the latest classification of antituberculosis drugs, RR/MDR-TB cases were treated with a regimen of five or six drugs (regimen 1), including one fluoroquinolone (ofloxacin, levofloxacin, or moxifloxacin), one second-line injectable drug (amikacin, kanamycin, or capreomycin), and two or three oral agents (including prothionamide, cycloserine, and para-aminosalicylic acid), with systematic addition of ethambutol and pyrazinamide, the duration of regimen 1 ranging from 18 to 20 months as per the WHO recommendations.^12^ Bedaquiline, introduced in Mexico in 2017, has been used nationwide by the Mexican National Tuberculosis Program since 2019. Since then, RR/MDR-TB cases have been treated at referral centers with three group A drugs-levofloxacin/moxifloxacin, bedaquiline, and linezolid-and one or two group B drugs-clofazimine or cycloserine-i.e., regimen 2 (Table 1). The use of clofazimine vs. cycloserine depends on whether there is central nervous system involvement, given that cycloserine has better cerebrospinal fluid penetration.^13^ The duration of regimen 2 was initially 18 months as per the WHO recommendations; however, after careful programmatic evaluation, it was reduced to a minimum of 15 months.^3^ ^,^ ^6^ Patients receiving either regimen underwent directly observed treatment. 

### Treatment monitoring

Patients underwent monthly follow-up visits during the intensive phase and every two months during the treatment maintenance phase. At each visit, blood tests were requested in order to assess adverse events. Since the addition of bedaquiline, a 12-lead electrocardiogram is also performed, and a sputum sample for culture is obtained in order to monitor treatment response. 

### Study population

All consecutive microbiologically confirmed RR/MDR-TB cases treated for at least 30 days with regimen 1 or regimen 2 were included. All selected cases had pulmonary involvement. 

### Statistical analysis

Regimen 1 and regimen 2 were compared in terms of efficacy and safety. The WHO definitions for treatment outcomes and adverse events were used. A bivariate analysis of variables (either categorical or numerical depending on their distribution) was conducted. Variables significantly associated with a successful outcome were considered for a multivariate logistic regression analysis including age, sex, HIV status, and type 2 diabetes mellitus (T2DM). 

All analyses were performed with the Stata statistical software package, version 13.0 (StataCorp LP, College Station, TX, USA). 

## RESULTS

Between 2010 and 2023, a total of 126 patients (101 patients at the INER and 25 at the *Hospital General de Tijuana*) met the inclusion criteria. Of those, 117 (92.8%) were culture positive at diagnosis, the remaining being diagnosed on the basis of a positive GeneXpert MTB/RIF test result for rifampin resistance. A total of 96 patients (76.2%) underwent drug susceptibility testing for fluoroquinolones. One hundred and twenty (95.2%) had RR/MDR-TB, with 6 (4.8%) showing additional resistance to a fluoroquinolone (Table 1). 

Thirty-nine patients received regimen 2, including bedaquiline and another two group A drugs (Table 2). Clofazimine was included in 37/39 (95%) cases, with 6 patients receiving additional cycloserine because of central nervous system involvement, all of them being coinfected with HIV. 

**Table 2:** Former tuberculosis treatment regimen (regimen 1) and the regimen that is currently used in Mexico (regimen 2): drugs and doses.

Variable	REGIMEN 1	REGIMEN 2
Fluoroquinolones		
Ofloxacin	600-800 mg	
Levofloxacin	750-1,000 mg	750-1,000 mg
Moxifloxacin	400-800 mg	400-800 mg
Second-line injectable drugs		
Amikacin	15-20 mg/kg	----------
Kanamycin	15-20 mg/kg	----------
Capreomycin	15-20 mg/kg	----------
Prothionamide	15-20 mg/kg	-----------
Cycloserine	10-15 mg/kg	10-15 mg/kg*
Ethambutol	15-25 mg/kg	
Pyrazinamide	25-35 mg/kg	
Bedaquiline	-------------	400 mg × 2 weeks 200 mg/3 times per week for 22 weeks
Linezolid		600 mg/day
Clofazimine		100 mg/day
Intensive phase of treatment	6-8 months	24 weeks
Treatment duration	18-20 months	15-18 months

*Used in 6 patients in the present study, all of whom had central nervous system involvement.

Regimens 1 and 2 were comparable for the variables reported in Table 1, the exception being that more patients receiving regimen 1 reported a history of previous tuberculosis treatment (primary regimen) and more patients receiving regimen 2 were living with HIV. Therefore, cavitary disease was more common in those patients (82.6% vs. 59%; p = 0.005), as were the related parameters (culture and sputum smear positivity). 

The prevalence of T2DM was high among drug-resistant cases^13^ in the sample as a whole, being = 57 (45.2%), with a median duration of 10 years [IQR, 7-15 years], although no difference was found between patients receiving regimen 1 and those receiving regimen 2 (Table 1). 

Success rates were not significantly different between the two groups of patients (p = 0.246); however, cases treated with the oral regimen including bedaquiline (regimen 2) had higher success rates (Table 3). Regimen 2 patients experienced a shorter time to culture conversion in comparison with regimen 1 patients (1.7 [1.0-2.1] vs. 2.2 [1.2-2.7] months; hazard ratio = 1.75; 95% CI, 1.08-2.83; p = 0.022). Although a history of T2DM was initially associated with a longer time to culture conversion, in the proportional hazards model, after adjustment for cavitary disease, T2DM, and HIV infection, the strength of the association increased (adjusted hazard ratio = 1.81; 95% CI, 1.11-2.95; p = 0.016; Table 4), and the presence of cavitary disease was associated with a longer time to culture conversion (adjusted hazard ratio = 0.57; 95% CI, 0.34-0.96; p = 0.036; Table 4). 

**Table 3:** Regimen 1 and 2 outcomes (bivariate analysis).^a^

	Regimen 1 (n = 87)	Regimen 2 (n = 39)	p
Positive outcome Cure Treatment completion	63 (72.4%) 59 4	32 (82%) 29 3	0.246
Negative outcome Loss to follow-up Failure Death	24 (27.6%) 12 4 8	7 (18%) 3 1 3	0.183
Intensive phase, months	7.0 [5.9-7.7]^b^	5.5 [5.2-5.5]^c^	0.0000
Time to culture negative status, months	2.2 [1.2-2.7]	1.7 [1.0-2.1]	0.0221

a Data presented as n, n (%), or median [IQR]. ^b^Data available for 66 patients. ^c^Data available for 16 patients.

**Table 4:** Hazard ratios for univariate and multivariate analyses.

	HR	95% CI	p	aHR*	95% CI
Sex	0.78	0.52-1.17	0.236	------	--------
T2DM	0.93	0.63-1.37	0.699	------	---------
HIV infection	0.62	0.22-1.71	0.355	------	---------
Cavitary disease	0.64	0.39-1.03	0.068	0.57	0.34-0.96
Regimen	1.75	1.08-2.83	0.022	1.81	1.11-2.95

T2DM: type 2 diabetes mellitus; HR: hazard ratio; and aHR: adjusted HR. *The adjusted model included a history of T2DM, HIV infection, and presence or absence of cavitary disease.

**Figure 1:**
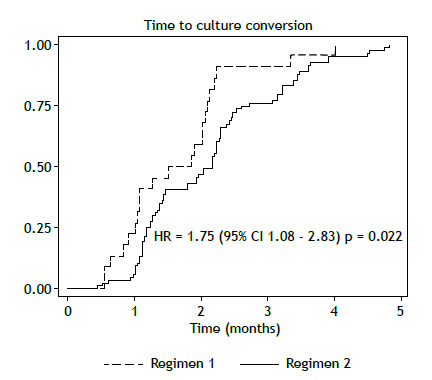
Time to culture conversion in tuberculosis patients treated with regimen 1 or 2. HR: hazard ratio.

Given that the patients who received the oral regimen had a faster sputum culture conversion (Figure 1), the length of the regimen was shortened on the basis of medical evaluation, the mean duration being 16.1 months [IQR, 15-17.3 months]. 

As can be seen in Table 3, a higher number of patients receiving regimen 1 experienced a negative outcome: loss to follow-up (12 vs. 3); treatment failure (4 vs. 1); or death (8 vs. 3). However, none of these outcomes was statistically significant between the two groups of patients. 

The median time elapsed between treatment initiation and loss to follow-up was 4.9 months [IQR, 2.1-6.6 months] for regimen 1 and 5.0 months [IQR, 3.4-6.4 months] for regimen 2. Two patients who had been lost to follow-up were later evaluated and remained bacteriologically negative. 

Adverse events are reported in Table 5, by regimen and type. Adverse events were the main reason why patients receiving regimen 1 decided to stop their treatment, whereas, among those receiving regimen 2, one could not be followed because of the COVID-19 pandemic; one had to move to another state; and one had gastrointestinal adverse events only. 

**Table 5:** Adverse events observed in tuberculosis patients enrolled to receive treatment regimen 1 or 2.^a^

	Regimen 1 (n = 87)	Regimen 2 (n = 39)	p
Hepatotoxicity	2 (2.3%)	4 (10.2%)	0.052
Nephrotoxicity	36 (41.4%)	2 (5.1%)	0.000
Ototoxicity	25 (28.7%)	0	0.000
Hypothyroidism	21 (24.1%)	0	0.001
Psychiatric disorder	14 (16.1%)	1 (2.5%)	0.029
Neuropathy	1 (1.1%)	12 (31%)	0.000
Myelotoxicity	0	4 (10.2%)	0.003
Skin reaction	6 (6.9%)	11 (28.2%)	0.001
QT prolongation	Not evaluated^b^	6 (15.4%)	(*not done*)

a Data presented as n (%). ^b^Before the introduction of the new drugs, patients were never evaluated for QT prolongation.

Patients treated with regimen 1 reported adverse events mainly related to the use of second-line injectable drugs: nephrotoxicity (an increase in serum creatinine ≥ 0.3 mg/dL) and ototoxicity (Table 5). Although a greater number of patients receiving regimen 2 developed hepatotoxicity [in 4 (10.3%)], there was no need to stop or modify the regimen. 

Among the patients treated with the oral regimen, most of the adverse events were related to linezolid, including neuropathy (clinically assessed) and myelotoxicity, the median time to an adverse event being 5.2 months [IQR, 4.1-8.75 months]. Only 5 patients had to stop the drug even when the linezolid dose was reduced to 300 mg. 

Among the patients treated with regimen 2, 6 (15.4%) experienced Fridericia-corrected QT interval prolongation ≥ 500 ms, the median time to this adverse event being 1.05 months [IQR, 1.05-2.7 months]. Bedaquiline had to be removed from the regimen in one case only; in another, the drug was reintroduced at a daily dose of 100 mg. 

Skin hyperpigmentation related to the use of clofazimine (regimen 2) was generally mild, being severe in 11 cases (28.2%); however, no patient reported this complaint. 

Patients treated with regimen 1 also experienced cutaneous adverse events (6.9%), mostly rash with or without pruritus (easily managed with ancillary medications), although one patient experienced drug rash with eosinophilia and systemic symptoms syndrome caused by levofloxacin. 

Adverse events related to second-line antituberculosis treatment were more common among T2DM patients receiving regimen 1 or regimen 2^14^ (Table 2). Among the patients treated with regimen 2, a history of T2DM was significantly associated with an increased risk of developing adverse events (neuropathy, myelotoxicity, hepatotoxicity, or QT prolongation; OR = 15.4; 95% CI, 2.73-87.29; p = 0.002). Notably, linezolid-associated neuropathy was more common among T2DM patients (3 vs. 9; p = 0.001). In a multivariate analysis adjusted for sex, age, and T2DM, the development of neuropathy remained associated with a history of T2DM (adjusted OR = 10.67; 95% CI, 1.72-62; p = 0.011). Among the patients receiving regimen 2, we found no difference in time to culture conversion between those with and those without T2DM.^15^

No relapses were reported by patients receiving regimen 1, whereas, among those receiving regimen 2, relapse could only be evaluated at one year, with 30/39 (77%) patients completing their treatment successfully. 

## DISCUSSION

The objective of the present study was to compare the former regimen including one fluoroquinolone and a second-line injectable drug (regimen 1) with the current regimen including the three group A drugs (regimen 2) in terms of efficacy and safety at two tuberculosis referral centers in Mexico. 

Several systematic reviews and meta-analyses have demonstrated the efficacy and safety of the addition of bedaquiline to tuberculosis treatment regimens, highlighting how the inclusion of this drug instead of second-line injectable drugs has enabled the development of fully oral and effective second-line regimens. 

The results of our study are different from those of a previous retrospective study conducted in Brazil,^16^ where a bedaquiline-containing regimen (similar to regimen 2 in our study but using terizidone instead of clofazimine) was associated with positive outcomes but no shorter time to culture conversion. In our study, despite a smaller sample size and a higher number of patients with T2DM, we observed similar success rates (and proportions of negative outcomes) between the two groups of patients. Notably, patients treated with an all-oral regimen including bedaquiline (regimen 2) had a shorter (nearly 50% shorter) time to culture conversion, thus potentially reducing tuberculosis transmission and treatment duration. 

Patients receiving either regimen 1 or 2 in the present study were similar for the main variables, with two notable exceptions. Regimen 1 patients more often had a history of previous tuberculosis treatment (81.6% vs. 62%; p = 0.015), probably due to the introduction of GeneXpert MTB/RIF in Mexico as an initial diagnostic tool in 2016, and were less likely to be living with HIV (3.4% vs. 16.6%; p = 0.006). 

T2DM is frequently associated with drug-susceptible and drug-resistant tuberculosis in Latin America, especially in Mexico.^14^ In our cohort, the prevalence of T2DM was high (44.6%) in comparison with that reported in other studies conducted in Latin America.^16^ Although T2DM has a negative effect on MDR-TB outcomes,^17^ we found no difference in outcomes between patients with or without T2DM, probably because of the effective management of T2DM at the two tuberculosis referral centers. However, a comprehensive evaluation of the two regimens must consider safety and tolerability. As previously described, patients receiving regimen 1 were mainly affected by nephrotoxicity, ototoxicity (related to second-line injectables drugs) and psychiatric disorders, all of which are commonly observed in T2DM patients. 

Patients who received regimen 2 in the present study were mostly affected by linezolid-related toxicity (neuropathy and myelotoxicity). Of all WHO group A drugs, linezolid is considered the most toxic, being responsible for major adverse events such as neuropathy (in 31% of patients), whereas myelotoxicity had a lower impact (9.5%). Tolerance to prolonged use of linezolid has been a significant limitation of new treatment regimens. The 600 mg/day dose used in our group of patients appeared to be the best tolerated, with fewer serious adverse events.^18^ ^,^ ^19^ In fact, linezolid is the drug for which therapeutic drug monitoring is strongly recommended^20^; unfortunately, it is not yet accessible globally, particularly in low- and middle-income countries, where the prevalence of drug-resistant tuberculosis remains elevated.^21^ In absence of therapeutic drug monitoring, close clinical follow-up is essential to identify early linezolid-related adverse events.^22^

Tolerance to linezolid is of paramount importance when using shortened regimens (including BPaL/BPaLM) to prevent frequent changes in the regimen. In the present study, the median time to a linezolid-related adverse event was five months; this means that linezolid was used at the full dose for a sufficient duration to ensure a good bactericidal activity, being then either reduced or removed from the regimen. In addition to the dose of linezolid, patient-specific variables such as preexisting comorbidities (e.g., T2DM) play a role in the development of neuropathy.^23^

When discussing the adverse events of fluoroquinolones, we must consider QT prolongation. This adverse event was not considered significant until the introduction of new and repurposed drugs such as bedaquiline, clofazimine, and delamanid. Among fluoroquinolones, moxifloxacin carries the greatest risk of QT prolongation and therefore a higher risk of serious ventricular arrhythmia^24^; this is the main reason why levofloxacin was preferred over moxifloxacin in regimen 2 (37 vs. 2 patients). QT prolongation (> 500 ms) has been reported in approximately 10% of cases of patients receiving bedaquiline-based regimens^25^ ^-^ ^27^; in our study, the prevalence of this adverse event was mildly higher (14.3%). Bedaquiline is considered safe; in one case only was the drug removed from the regimen, whereas, in another, it was reintroduced at a daily dose of 100 mg. 

Within regimen 2, clofazimine has been reported to cause skin hyperpigmentation in approximately 50% of cases.^28^ In our study, severe hyperpigmentation was observed in only 11 cases (28.2%), although, interestingly, no patient complained about this adverse event. 

The similarities and equal distribution of features potentially hampering treatment outcomes between the two groups (history of previous tuberculosis treatment and HIV coinfection) can be considered a strength, as can the programmatic perspective from two of the main referral centers in a priority country such as Mexico. We were able to evaluate the adverse events of the main drugs from a real-life perspective in Mexico. However, although the information collected was detailed, the retrospective nature of the study is a limitation, as is the lower sample size for regimen 2. Furthermore, despite the efforts of the staff of the two referral centers, relapse could not be assessed in all patients. 

The use of new and repurposed drugs enabled a shift to an oral and effective regimen in Mexico, although toxicity to linezolid requires strict patient monitoring. Recently, the WHO introduced an all-oral nine-month regimen including bedaquiline, linezolid, levofloxacin, clofazimine, and pyrazinamide to treat patients with levofloxacin-sensitive RR/MDR-TB strains.^29^ ^,^ ^30^ This drug regimen of four or five drugs is similar in Mexico, although without pyrazinamide; it appears to be highly bactericidal (given that most cases tested negative by the first month), offering a safer and effective treatment option without adding additional toxicity related to pyrazinamide. Consequently, extending the regimen to 18-20 months is generally unnecessary. Further studies are required to confirm these findings. 

In summary, oral regimens appear to be effective, although toxicity to linezolid requires strict patient monitoring. 
